# Metagenomics: A foundling finds its feet.

**DOI:** 10.4056/sigs.1213842

**Published:** 2010-10-27

**Authors:** Jack A. Gilbert, Folker Meyer, Dawn Field, Lynn M. Schriml, George M. Garrity

**Affiliations:** 1 Argonne National Laboratory, Argonne, IL, U.S.A.; 2 Department of Ecology and Evolution, University of Chicago, Chicago, IL, U.S.A.,; 3 Computation Institute, University of Chicago, Chicago, IL, U.S.A.,; 4 NERC Centre for Ecology & Hydrology, Wallingford Oxford, UK.; 5 University of Maryland School of Medicine, Baltimore MD, USA; 6 Standards in Genomics, Michigan State University, East Lansing, MI USA

When Jo Handelsman first coined the term Metagenomics in 1998 [1], who would of foreseen the rapid progression of a clonal-based research technology handling hundreds of thousands of base pairs to the goliath sequencing efforts that are routinely applied today? Metagenomics, or community-based genome sequencing and analysis, has come of age. In Greek, ‘meta’ refers (in this context) to ‘being beyond’, and it can truly be said that we have now gone beyond the genome. With the combined application of the two dominant next-generation sequencing platforms, 454-pyrosequencing and Illumina-Solexa, it is now possible to sequence an environmental metagenome and reassemble whole genomes from the small sequencing fragments. Within this context we introduce the short-metagenome reports for Standards in Genomic Sciences. The short genome reports were developed in response to the sudden and rapid increase in the quantity of genome sequences being sequenced, combined with the inertia exhibited by society and commercial publishers to provide a dedicated outlet for these genomes. It was viewed as essential that these genomes be published with their associated metadata so that any researcher could access this information in one location. Now that metagenomics is reaching a similar position, with hundreds of new metagenomes being produced each month, it is time that a place be provided where the community can go to find these valuable datasets along with their associated environmental characteristics, experimental parameters, sequencing technology information, and analysis pipelines.

Unlike genome sequencing which has a defined end point, a completed circularized chromosome(s), a metagenome currently has no such end. This has much to do with issues surrounding the vast complexity of microbial communities. To date no metagenomic reports, either single gene or shotgun, have been produced that fully characterize every base pair in an environmental sample. This is unsurprising as most environmental sampling volumes (e.g. aquatic = >1 L; soil = >1 g) contain in excess of 3-4 quadrillion (1 x 1015) base pairs of DNA. Currently this would cost approximately $30 Million to sequence to one fold coverage, providing > 1000 × coverage for abundant species, and lower coverage the further we go down the rank abundance curve. Importantly this kind of analysis could enable us to start asking questions regarding our ability to differentiate closely related microbial species. The pan-genome concept describes the shared genetic content between strains of a given species. Currently, when we attempt to assemble a metagenome we tend to average out small variations that could be ascribed to the pan-genome in order to merge the pan-genome into a single genome. Deep sequencing will enable us to provide more and more resolution when assembling genomes, for example if we can resolve one complete genome when we sequence 10 billion base pairs, we may be able to resolve two strains with a very similar genome (say 3-4% varience) if we sequence 20 billion base pairs (depending on the abundance of the strain), likewise we if sequence 100 billion base pairs we could start to resolve a number of different genomes and potentially different strains for each genome – the more you sequence the more you see. This will enable us to better understand the role of genomic variation within a population in enabling adaptation to environmental change.

Further exploration of microbial ecosystems must be based on our current understanding of each sampling site. To do this we must know what work was done and exactly how it was done. It is vital that the community realizes that as we move forward we must work together. Standardization of data associated with a metagenome will enable future researchers to explore connectivity between samples and determine how and why microbial groups exist in specific locations. Only now are we truly capable of sequencing whole communities. The limiting factor, cost, will decrease, further democratizing this technology. Even so, mega-sequencing projects are still important; to showcase what can be achieved through the use of centralized resources. The global ocean sampling project of the J. Craig Venter Institute is an ideal example of a project that galvanized a research area. Future projects will aim to explore communities on an even grander scale. We will soon be in the terabase-sequencing era, quickly followed by the petabase-sequencing era – how we utilize this data will depend solely on how well we collaborate as a community. Visions such as the Earth Microbiome Project (www.earthmicrobiome.org) are working towards such goals, through production of a global environmental sample database (GESD), which will catalogue all the samples and associated metadata, which can be found in researchers’ storage across the globe. Once this information is available it can be used to direct sequencing efforts to best explore and describe microbial diversity, and consequently protein diversity, on our planet. Of course we must all start somewhere, and provision of currently available data in a standard format is a good place to start.

It is very important that we catalogue every metagenome produced in a standardized format with a minimal suite of metadata associated with the report. This will enable us to start exploring concepts such as strain variation and environmental consequence in broader and more deterministic fashion. Importantly, this report format is not limited to metagenomes, but is adaptable for metatranscriptomic, and potentially metaproteomic reports. Let no information produced by the community fall through the gaps. This centralized resource will ensure that all the information is readily accessible and can be found in one place.

## References

[1] Handelsman J Rondon MR Brady SF Clardy J Goodman RM Molecular biological access to the chemistry of unknown soil microbes: a new frontier for natural products. Chem Biol 1998; 5:245-249 10.1016/S1074-5521(98)90108-9 9818143

